# Will Spam Overwhelm Our Defenses? Evaluating Offerings for Drugs and Natural Health Products

**DOI:** 10.1371/journal.pmed.0040274

**Published:** 2007-09-18

**Authors:** Peter Gernburd, Alejandro R Jadad

## Abstract

At least one-third of spam e-mails include offers of health products, according to this new study.

Spam, the term used to describe unsolicited e-mail received from an unknown sender without expressed consent of the recipient [1], has become a major problem for communications through the Internet. Since the delivery of the first spam message in 1978 [2], the volume of spam has grown tremendously. In January 2005, the percentage of all e-mails, worldwide, identified as spam was estimated at 73% [3].

Spammers (the name given to those who deliver spam) are motivated by the fact that a sufficiently large proportion of e-mail users are lured by their offers. In 2003, it was estimated that one-third of e-mail users had clicked on a Web link in a spam message, and 7% had ordered a product [4].

Despite the growing importance of spam for many industries and the public's strong interest in online health information, very little seems to be known about the relationship between spam and human health. Although it has been estimated that 10% of spam includes health-related offers [5], a search of MEDLINE on March 21, 2007, using the terms “spam,” “unsolicited e-mail,” or “junk e-mail” (limited to titles and abstracts, and using the search terms with and without a hyphen), failed to yield citations of original systematic studies on health-related spam. With this study, to our knowledge the first of its kind, we hope to start filling this gap.

## Methods

**Sampling frame.** Three accounts, unfiltered for spam, were used to collect the data from November 1 to November 30, 2006. All accounts were based in Ontario, Canada, and were active for at least six months before data collection started. One of the accounts, which we labeled research, belonged to a member of the Centre for Global eHealth Innovation (http://www.ehealthinnovation.org/); another, labeled commercial, belonged to a commercial provider of communication services (Mountain Cablevision; http://www.mountaincable.net/); and one acted as a control and was set up to collect unsolicited messages.

**Selection criteria.** A message was regarded as spam if it:

- had been sent by an unrecognized source;
- originated from a mailing list with which the recipient had no previous relationship; and
- contained words with altered spelling in the subject line or included explicit unsolicited commercial offers [6].

**Summary Points**

- Spam, or unsolicited e-mail received from an unknown sender, now accounts for the largest proportion of all messages delivered online.
- Little is known about health-related spam and the spammers behind it.
- This study shows that it is possible to purchase products purported to be prescription drugs and controlled substances, across traditional national and legal boundaries, with one-third of our attempts to do so being successful.
- Buyers should be fully aware that it may not be possible for them to hold spammers accountable for any claims made in their messages, or to get protection from illegal activities resulting from disclosure of personal or financial information to spammers.
- Spammers are challenging our traditional regulatory, licensing, and law enforcement frameworks, and even threatening their relevance.

**Data extraction.** All messages deemed to be spam were stored as a common dataset, and were labeled as health-related if they included offers of interventions that could modify a physical, cognitive, behavioral, or emotional state in humans (e.g., medications, natural health products, devices, or professional services).

All health-related messages that contained an active Web link (a URL within a message that led to a working Internet location), and that had been received between November 23 (Thanksgiving day) and November 30, were analyzed for the extraction of the following information:

- Sender details: The name and Web address of the organization that generated the message, the country of origin, and contact information
- Product offered: Product name, claim of benefit (yes/no/unclear), and mention of potential adverse effects (yes/no/unclear)
- Costs and payment options: Minimum purchase price, shipping fees, methods of payment
- Return policy: Refund (full or partial), product exchange, or not specified

All messages with active Web links were analyzed to determine whether they included unique offers. A unique offer, in our research, was considered for analysis if the active web link in the message pointed to a nonduplicate Internet location or if the message contained unique sender details.

**Product purchase.** One product was ordered from each unique offer on December 21, 2006, using a Visa card and a Post Office Box. When the offer included more than one product, an item listed was selected randomly for purchase, drawing lots.

## Findings

The study accounts received 4,153 unsolicited messages (82% of the total messages received) during the month of November 2006. Health-related spam accounted for 32% (n = 1,334). Nineteen messages had active Web links during the last week of the month. These messages had been received a total of 143 times by the accounts. Of these, 73% (n = 104) had been sent from the United States, 16% (n = 23) from China, and 5% (n = 8) from the Democratic Republic of the Congo (Table 1). Only 58% of the active links in health-related messages received during the first week remained active by end of the second week; while 26% were active by the end of the month. Thirteen of the 19 messages included offers of prescription drugs and six included offers of natural health products. None included services or devices. Three of the messages offered free shipping of products, while the rest charged between US$9.00 and US$19.95. Only half of the messages included statements on refund of products.

**Table 1:**
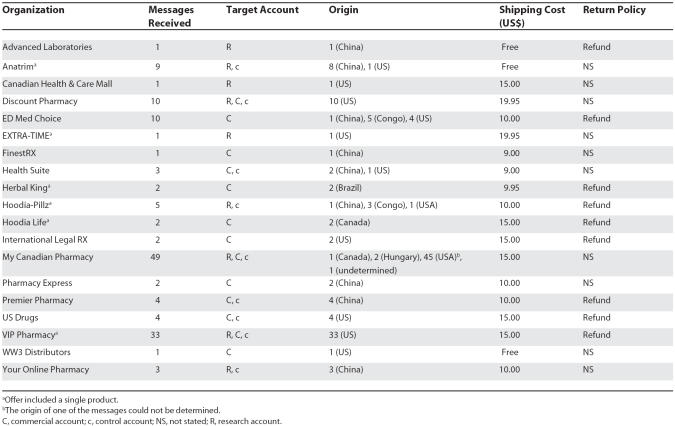
Spammers: General Characteristics

The 19 messages contained 27 unique offers, which were used to order products (13 prescription drugs and six natural health products). All of the sites from which the products were ordered included claims of benefit and warned about potential adverse effects, which were so diverse across messages that it was not possible to match them with approved lists. During the order process, four Web sites stopped working after the credit card information was submitted. No further information was provided on whether the transaction was successful. Thirteen sites did not process the order, but received all of the information provided.

As of July 21, 2007, nine orders had been delivered, including five prescription drugs and four natural health products (Tables 2 and 3), with no evidence of attempts by spammers to abuse the credit card information given to them, except for one undelivered product for which the order was successfully completed.

**Table 2:**
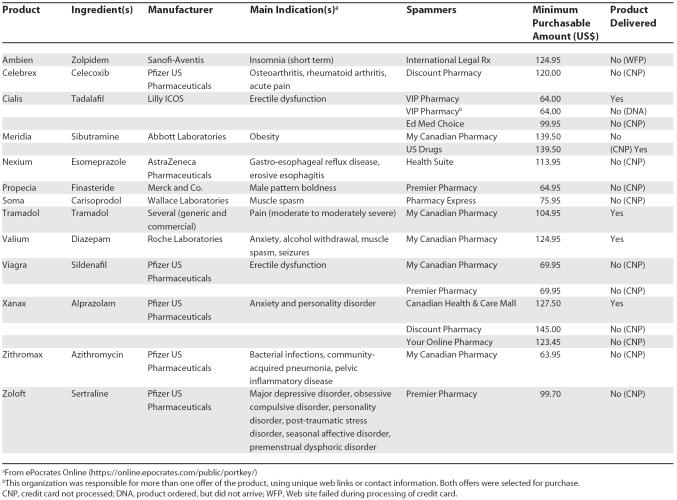
Prescription Drugs Ordered from Spammers

**Table 3:**
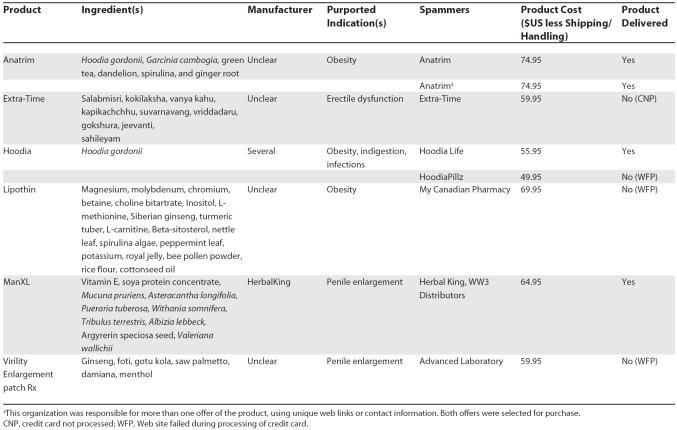
Natural Products Ordered from Spammers

## Discussion

We approached and completed this project with trepidation. We knew that we would be interacting with organizations operating at the margins of the law or clearly outside it. It was not easy to get approval from university administrators for a project that could include ordering products from unknown and unverified sources, and which could lead to illegal trade of substances, or costs associated with items for the enhancement of sexual function. It was unsettling to order the products, not knowing whether we were contacting undercover law enforcement agents (to whom it would be difficult to explain the research nature of the project) or fronts for unscrupulous criminals who could abuse the financial information or even steal our identities. We believe that the effort and stress were worthwhile.

The study confirms that spam is responsible for at least two-thirds of e-mail traffic [2,4], and suggests that at least one-third of the unsolicited messages include offers of health products. Our findings show that it is possible to purchase products purported to be prescription drugs and controlled substances, across traditional national and legal boundaries, and that about one-third of the attempts to do so will be successful. This is a much higher rate than the previously reported 17% for generic attempts to purchase products from spammers [6]. It is unclear whether this is a reflection of an increased level of confidence of spammers over the years, or a specific phenomenon related to health-related products. The volume of messages does not seem to relate to the level of outward e-mailing activity of the spam recipient, as the account we set as control received a similar amount of unsolicited material.

Our results also underscore the elusive nature of spammers and their activities. Spam messages are delivered through a rich array of servers, links, and accounts; we found that the group of accounts we identified had a half-life of around two weeks (i.e., it took two weeks for half of the accounts to become inactive). Buyers should be fully aware that it may not be possible for them to hold spammers accountable for any claims made in their messages, or to get protection from illegal activities resulting from disclosure of personal or financial information to spammers.

Although there is concern about the delivery of prescription drugs from Canadian online pharmacies to US-based customers, the bulk of the spam received by the Canada-based accounts used in the study was generated in the United States. As with online pharmacies [7,8], highly mobile spammers are certainly challenging our traditional regulatory, licensing, and law enforcement frameworks, and even threatening their very relevance.

Because of the short life of the active links, it was difficult to take action against the spammers. By the time the products were received, in each case, the spammers had become virtual ghosts, precluding any meaningful effort to report them to custom officers, local or regional police forces, national law enforcement agencies (the Federal Bureau of Investigations in the United States and the Royal Canadian Mounted Police in Canada), or organizations such as the US Department of Health, the US Federal Drug Administration, Health Canada, or INTERPOL. We are currently searching and contacting, systematically, the Web sites of these organizations, trying to determine the best course of action regarding health-related spam. This is proving challenging, as most of the above bodies seem to lack a clear strategy to deal with spam-related issues.

Our study had limitations, which we hope will be overcome in future efforts to understand health-related spam. Perhaps if we had included a larger number of accounts and focused on a longer study period, we would have increased the generalizability of the results or produced more evidence about the risks associated with pursuing offers from spammers. However, several months following the provision of financial information online, we have no indication of abuse. Instead, we have nine products that seem to match what was offered. We are also conducting a study to determine whether the pharmaceutical products we received are genuine, fakes, or substandard. We hope to report some new insights on these issues in the very near future.

Hope and greed are two powerful human motivators. As the number of people who turn to the Internet looking for solutions to health or lifestyle problems increases, merchants will rise to the occasion, matching the demand. As this study has shown, current regulatory, legal, and geographic boundaries may be unable to contain the flow of products across the world. Despite its potential for harm, it could be argued that spam is heralding a new era in which, thanks to the power of the Internet, those boundaries will be re-assessed, redrawn. or even eliminated.
